# COVID-19-related stigma among the non-infected general population from Iran

**DOI:** 10.1192/j.eurpsy.2022.1230

**Published:** 2022-09-01

**Authors:** A.H. Nadoushan, M. Faghankhani

**Affiliations:** 1 Iran University of Medical Sciences, Psychaitry, Tehran, Iran; 2 Iran University of Medical Sciences, Mental Health Research Center, Tehran, Iran

**Keywords:** Iran, stigma, Covid-19

## Abstract

**Introduction:**

COVID-19-related stigma has been raised as a crisis since the beginning of the pandemic. We intended to develop a valid and reliable questionnaire to measure COVID-19-related stigma, attributed by the non-infected general population, and applied it in Tehran from September to October 2020.

**Objectives:**

This study measures the COVID-19-related stigma attributed by the non-infected general population using a valid and reliable questionnaire specific to COVID-19-related stigma.

**Methods:**

A preliminary questionnaire with 18 items was developed. The score ranged from 18 to 54 while the higher score indicating a higher level of COVID-19-related stigma. Out of 1064 randomly recruited Tehran citizens without a history of COVID-19 infection, 630 participants, who completely responded to the questions on a phone call, entered the study.

**Results:**

The content validity was established with a scale content validity index of 0.90. Item CVI and Item content validity ratio were higher than 0.78 for all items. Internal consistency was confirmed with Cronbach’s alpha of 0.625. Exploratory factor analysis revealed seven latent variables, including “blaming and penalty-seeking behavior”, “social discrimination”, “dishonor label”, “interpersonal contact”, “spreading rumors and myths”, “overvalued idea”, and “apathy toward the patients”. The mean (SD) of the score was 25.1(4.71) in our study. 86.8% of participants reported a low level of stigma with a score below 31. 13.2% of them demonstrated a moderate level of stigma, and none of the participants showed a high level of stigma.

**Conclusions:**

we found a low level of stigmatizing thoughts and behavior in Tehran, which may be due to social desirability bias.

**Disclosure:**

No significant relationships.

